# A Substantial Fraction of Barley (*Hordeum vulgare* L.) *Low Phytic Acid* Mutations Have Little or No Effect on Yield across Diverse Production Environments

**DOI:** 10.3390/plants4020225

**Published:** 2015-04-29

**Authors:** Victor Raboy, Kevin Peterson, Chad Jackson, Juliet M. Marshall, Gongshe Hu, Hirofumi Saneoka, Phil Bregitzer

**Affiliations:** 1 USDA-ARS Small Grains and Potato Research Unit, 1600 South 2700 West, Aberdeen, ID 83210, USA; E-Mails: Peterson.kevin1@mayo.edu (K.P.); Gongshe.hu@ars.usda.gov (G.H.); phil.bregitzer@ars.usda.gov (P.B.); 2 Idaho Falls Research and Extension Center, University of Idaho, 1776 Science Center Drive, Suite 205, Idaho Falls, ID 83402, USA; E-Mails: chadj@uidaho.edu (C.J.); jmarshall@uidaho.edu (J.M.M.); 3 Graduate School of Biosphere Science, Hiroshima University, 1-4-4 Kagamiyama, Higashi-Hiroshima 739-8528, Japan; E-Mail: saneoka@hiroshima-u.ac.jp

**Keywords:** barley, *Hordeum vulgare*, low phytic acid, grain yield

## Abstract

The potential benefits of the *low phytic acid* (*lpa*) seed trait for human and animal nutrition, and for phosphorus management in non-ruminant animal production, are well documented. However, in many cases the *lpa* trait is associated with impaired seed or plant performance, resulting in reduced yield. This has given rise to the perception that the *lpa* trait is tightly correlated with reduced yield in diverse crop species. Here we report a powerful test of this correlation. We measured grain yield in lines homozygous for each of six barley (*Hordeum vulgare* L.) *lpa* mutations that greatly differ in their seed phytic acid levels. Performance comparisons were between sibling wild-type and mutant lines obtained following backcrossing, and across two years in five Idaho (USA) locations that greatly differ in crop yield potential. We found that one *lpa* mutation (*Hvlpa*1-1) had no detectable effect on yield and a second (*Hvlpa*4-1) resulted in yield losses of only 3.5%, across all locations. When comparing yields in three relatively non-stressful production environments, at least three *lpa* mutations (*Hvlpa*1-1, *Hvlpa*3-1, and *Hvlpa*4-1) typically had yields similar to or within 5% of the wild-type sibling isoline. Therefore in the case of barley, *lpa* mutations can be readily identified that when simply incorporated into a cultivar result in adequately performing lines, even with no additional breeding for performance within the *lpa* line. In conclusion, while some barley *lpa* mutations do impact field performance, a substantial fraction appears to have little or no effect on yield.

## 1. Introduction

The potential benefits of the *low phytic acid* (*lpa*) crop seed phenotype, in the contexts of human and animal nutrition and in the context of management of phosphorus (P) in animal agricultural production, are well documented [1]. However, there is a commonly encountered view that this seed trait is conditionally associated with reduced seed performance and reduced seed yield. This view often reflects sound science since in many cases the *lpa* trait is associated with impaired seed or plant performance [2]. However, many studies reporting on field performance of *lpa* genotypes present relatively limited data using lines obtained following limited or no breeding, which are often evaluated in a growth chamber or greenhouse bench, or one or a few field locations or replications over years. This limited experimental approach can also lead to the opposite but equally problematic and often not well-supported conclusion that a given *lpa* mutation has little or no impact on yield [3]. Further, comparisons are often made between lines that greatly differ in genetic background other than the *lpa* loci. Even comparisons made between a wild-type (for seed phytic acid) cultivar and an *lpa* mutation isolated following mutagenesis of that cultivar, or following most transgenic approaches [4], are less than optimal. Many genetic differences that can impact field performance, other than the *lpa* alleles, may exist between these types of lines. The strongest test of the relationship between the *lpa* trait and any other trait, whether it be yield or nutritional quality, is comparisons between sibling near-isogenic lines, obtained following sufficient backcrossing, and following selection for field performance in fixed genotypes that are either homozygous wild-type or homozygous mutant. Such sibling near-isogenic lines are genetically similar at most loci other than the *lpa* loci, so any differences observed in field performance or nutritional quality can validly be attributed to the different *lpa* locus alleles.

Here we report the results of what we believe is, to date, the most robust field evaluation of *lpa* variants of any crop. Six independently isolated barley (*Hordeum vulgare* L.) *lpa* mutations, representing lesions mapped to four different chromosomes therefore representing a minimum of four loci, were backcrossed to the cultivar (*cv.* Harrington) in which they were initially induced and isolated. For each mutation a set of near-isogenic sibling lines were then established, each set consisting of one mutant and one wild-type line. Field trials were then conducted over two years at five locations with widely divergent production conditions, providing a robust test of performance across environments.

## 2. Results

### 2.1. Barley Low Phytic Acid Mutations Selected for Isoline Development

**Figure 1:**
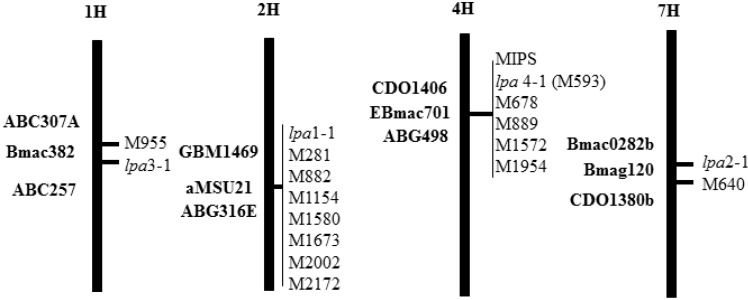
Chromosomal position of 17 barley *low phytic acid* mutations and the barley genome’s single *myo*-Inositol-3-P_1_ synthase (MIPS) gene. Map positions are approximate and only meant to be illustrative of linkage groups. As indicated, all mutations fell into four linkage groups, but allelic or non-allelic relationships within each linkage group are not yet known. The molecular markers used in mapping are on the left of each chromosome. The *lpa* mutations comprising each linkage group are on the right of each chromosome.

A screen of sodium azide-induced mutations in barley for the “high seed inorganic P” phenotype associated with *lpa* mutants had identified 23 putative mutants, of which 20 were selected for initial seed P phenotype evaluation (Supplementary Methods and Supplementary Table 1), and initial visual evaluation of plant performance. These 20 were grown in a nursery in 2004 in Aberdeen, Idaho, and following grain harvest, assayed for whole grain P fractions. Of these all but one displayed the low grain phytic acid trait (Supplementary Table 1). The one exception was *Hv-*MAZ423, which appears to be a high grain total P mutant in which all grain P fractions, including inorganic P, are elevated, as compared with wild-type. This putative high grain total P mutant is the subject of separate, ongoing study. Of the remaining 19 mutants, a total of 17 have been chromosomally mapped to date (Figure 1; Supplementary Tables 2 and 3).

Previous studies focused on four mutations that appeared to represent the range of seed P phenotypes observed, and that mapped to three linkage groups: *Hvlpa*1-1 (chromosome 2H), *Hvlpa*2-1 (chromosome 7H), *Hvlpa*3-1 (chromosome 1H), and *Hvlpa*-M955 (chromosome 1H). Based on initial observation of adequate plant performance, and to add mutations mapping to new chromosomal sites, for the present study we selected two additional mutations: *Hvlpa-*M593 and *Hvlpa*-M640. Initial quantitative analyses (Supplementary Table 1) indicated that *Hvlpa-*M593 conditioned a ~53% reduction in seed phytic acid, and that *Hvlpa*-M640 conditioned a ~47% reduction, but in the case of *Hvlpa*-M640, the effect appeared to be endosperm-specific.

Segregation analyses conducted during mapping indicated that these additional two mutants displayed an inheritance pattern of recessive alleles at single gene (Supplementary Table 2). Linkage analyses (Supplementary Table 3) revealed that *Hvlpa*-M593 mapped to chromosome 4H, defining a new barley *lpa* locus. Therefore hereafter, *Hvlpa*-M593 will be referred to as *Hvlpa*4-1. *Hvlpa-*M640 mapped to chromosome 7H, linked to *lpa*2-1. However, the allelic relationship if any between *lpa*2-1 and *lpa*M640 on chromosome 7H, or between *lpa*3-1 and *lpa*-M955 on chromosome 1H, is not known at present and is the subject of ongoing study. Thus these six mutations represent a total of four linkage groups and a minimum of four loci.

### 2.2. Confirmation of Isoline Seed Phytic Acid Phenotype

The seed inorganic P test was used for routine genotyping (homozygous wild-type *versus* homozygous mutant) of *lpa* isolines used in this study (Supplementary Figure 1 and Experimental Section). However, to provide a more in-depth confirmation of the seed P and phytic acid P phenotype of grain produced by these lines when grown at the locations used here, a quantitative analysis was conducted with grain samples from test plots at two 2011 locations, Aberdeen and Soda Springs (Table 1). These two locations were selected for this analysis because they represent the extremes in yield potential over all locations in this study (see below). This analysis confirmed that when grown in these specific locations, the predicted *lpa* phenotype of each mutant line (based on previous analyses such as given in Supplementary Table 1) was observed. Apparent reductions in grain phytic acid P, as compared with levels observed in grain produced by sibling wild-type lines, ranged from a low of ~22% in *Hvlpa*2-1, to the moderate reductions of ~35% in *Hvlpa*4-1, ~40% in *Hvlpa*-M640 and ~48% in *Hvlpa*1-1, to the more substantial reductions of ~57% in *Hvlpa*3-1 and ~80% in *Hvlpa-*M955. These are referred to as “apparent reductions” since the method used here probably overestimates phytic acid by 5% to 10%, an inflation resulting from reading components of the “extraction matrix” as phytic acid P when using this method. The best illustration of this is the data for *Hvlpa*-M955. Previous analyses of the grain phytic acid levels in this mutant using more accurate methods consistently indicated that the reduction in phytic acid P in this mutant, as compared with wild-type, is >90%. This analysis also confirmed that with one exception, *Hvlpa*1-1, the *lpa* mutations did not greatly impact grain total P: the reductions in grain phytic acid P were largely matched by molar-equivalent (in terms of P) increases in grain inorganic P. In the case of *Hvlpa*1-1, this analysis also confirmed that homozygosity for the mutation causes a reduction in whole-grain total P of 15% to 20% (Table 1). 

### 2.3. Yield Evaluations

To provide the most valid statistical approach to test for differences between sibling near-isogenic lines, the Analysis of Variance of the grain yield data presented in Table 2 was conducted using only the data for the six isoline pairs. The greatest variation observed was due to the main effects of Year and Location and the interaction of Year × Location. The large main effect of Year is clearly seen in the greater overall productivity in 2011 as compared with 2010; overall mean grain yield in 2011 (5.47 ± 0.28 Mg·ha^−1^) was ~20% greater than that observed in 2010 (4.52 ± 0.22 Mg·ha^−1^) (Table 3 and Table 4). The large main effect of Location is also clear in the data, and critical to the main objective of this study. Clear and large differences in overall mean grain yield per location, a measure of location yield potential, were observed in both years and the location ranking were similar in both years (from highest overall yield to lowest): Aberdeen > Idaho Falls > Rupert > Ashton > Soda Springs. The interaction effect of Year × Location was largely due to the very large difference in overall yield at one location, Ashton, where the yield in 2010 (2.43 ± 0.21 Mg·ha^−1^) was only 40% of that observed in 2011 (5.96 ± 0.32 Mg·ha^−1^). The large differences in productivity observed across the two years and five locations provides a powerful test of the impact on yield of an *lpa* mutation, the primary objective of this study.

**Table 1:** Seed total phosphorus (Total P), phytic acid P and inorganic P (P_i_) in pairs of sibling barley near-isogenic and cultivar controls grown in Aberdeen and Soda Springs, ID, in 2011.

Isoline Pair	Genotype	Idaho Field Location					
		Aberdeen			Soda Springs		
		Total P	Phytic Acid P	Inorganic P	Total P	Phytic Acid P	Inorganic P
**mg·g^−1^**							
1	WT	2.96	2.86	0.29	3.10	2.62	0.26
1	*Hvlpa*1-1	2.52	1.51	1.05	2.44	1.35	0.83
2	WT	2.86	2.47	0.24	3.09	2.75	0.25
2	*Hvlpa*3-1	2.76	1.10	1.45	2.88	1.15	1.66
3	WT	2.78	2.32	0.22	3.12	2.52	0.24
3	*Hvlpa-*M955	2.91	0.53	2.12	3.33	0.51	2.21	
4	WT	2.75	2.38	0.23	2.93	2.54	0.25	
4	*Hvlpa*2-1	2.93	1.83	0.67	3.18	1.99	0.90	
5	WT	3.10	2.49	0.39	2.98	2.59	0.27	
5	*Hvlpa*4-1	2.91	1.53	1.18	3.31	1.76	1.34	
6	WT	2.86	2.44	0.26	3.15	2.69	0.26	
6	*Hvlpa*-M640	2.98	1.61	0.80	3.36	1.51	1.32	
**Cultivar Controls**								
Harrington		2.73	2.37	0.26	3.07	2.59	0.30	
Baronesse		2.89	2.41	0.26	3.12	2.65	0.28	
Conrad		3.01	2.52	0.19	3.49	3.10	0.31	
Std. Dev. of Mean		0.12	0.14	0.07	0.15	0.14	0.05	
LSD *p* = 0.05		0.34	0.41	0.20	0.43	0.39	0.15	

**Table 2:** Analysis of variance of yield of twelve isolines grown in five locations in Idaho in two years.

Source of Variation	*df*	Mean Square	*F* Value	Prob. > F
Model	132	10.20	28.82	<0.0001
Year	1	97.24	274.70	<0.0001
Location	4	224.79	635.05	<0.0001
Year × Loc	4	61.43	173.54	<0.0001
Rep (Loc)	15	0.65	1.85	0.03
Isoline	11	3.70	10.45	<0.0001
Year × Line	11	0.22	0.62	0.82
Loc × Line	44	0.73	2.06	0.0002
Year × Loc × Line	42	0.47	1.31	0.11
Residual Error	330	0.35		

**Table 3:** Yield of barley *lpa* near-isogenic lines and three cultivar controls when grown in five locations in Idaho, 2010. Data represent means of four replicates at each location.

Isoline Pair	Genotype	Idaho Location					Line Mean
		Aberdeen	Ashton	Idaho Falls	Rupert	Soda Springs	
**Grain Yield (Mg·ha^−1^)**							
1	WT	6.04	2.52	6.15	6.20	2.13	4.61
1	*Hvlpa*1-1	6.61	2.78	6.57	5.47	2.00	4.69
2	WT	5.83	2.37	5.84	6.20	2.22	4.49
2	*Hvlpa*3-1	6.17	2.03	5.71	5.15	1.88	4.19
3	WT	5.72	2.59	5.98	5.45	2.80	4.60
3	*Hvlpa-*M955	5.27	1.88	5.71	4.74	1.47	3.81
4	WT	6.48	2.34	6.15	5.89	2.41	4.91
4	*Hvlpa*2-1	6.51	2.59	5.98	5.23	1.81	3.86
5	WT	6.14	2.32	6.45	5.71	2.52	4.63
5	*Hvlpa*4-1	6.06	2.30	6.74	5.45	1.96	4.50
6	WT	6.59	2.30	6.77	5.89	2.10	4.73
6	*Hvlpa*-M640	6.30	2.40	6.45	4.44	1.61	3.58
**Cultivar Controls**							
Harrington		6.38	2.42	5.54	5.54	2.22	4.42
Baronesse		7.17	3.57	6.81	7.45	2.59	5.52
Conrad		7.06	2.37	6.08	6.91	2.05	4.89
Location Mean ± SE		6.29 ± 0.34	2.43 ± 0.21	6.19 ± 0.29	5.78 ± 0.22	2.10 ± 0.19	4.52 ± 0.22
LSD *p* = 0.05		0.98	0.59	0.83	0.63	0.55	0.64

**Table 4:** Yield of barley *lpa* near-isogenic lines and three cultivar controls when grown in five locations in Idaho, 2011. Data represent means of four replicates at each location.

Isoline Pair	Genotype	Idaho Location					Line Mean
		Aberdeen	Ashton	Idaho Falls	Rupert	Soda Springs	
**Grain Yield (Mg·ha^−1^)**							
1	WT	6.51	5.79	6.10	5.22	3.05	5.33
1	*Hvlpa*1-1	7.35	6.69	5.57	5.57	3.25	5.68
2	WT	6.80	6.15	4.93	4.98	3.55	5.37
2	*Hvlpa*3-1	6.98	5.32	4.96	5.01	2.78	5.01
3	WT	6.40	6.34	5.57	4.84	3.59	5.35
3	*Hvlpa-*M955	5.07	4.83	4.86	4.61	2.47	4.37
4	WT	7.03	6.32	5.52	5.10	3.42	5.48
4	*Hvlpa*2-1	7.51	4.57	6.27	5.03	2.78	5.23
5	WT	7.80	6.37	5.96	5.25	3.44	5.76
5	*Hvlpa*4-1	6.95	5.84	5.27	5.88	2.99	5.51
6	WT	7.29	6.01	5.40	5.48	3.20	5.48
6	*Hvlpa*-M640	6.98	5.03	5.93	4.95	2.47	5.21
**Cultivar Controls**							
Harrington		6.96	6.27	5.69	5.30	3.17	5.44
Baronesse		8.61	7.13	6.86	6.08	4.05	6.55
Conrad		8.00	6.71	6.64	6.74	3.03	6.22
Location Mean ± SE		7.08 ± 0.37	5.96 ± 0.32	5.69 ± 0.29	5.34 ± 0.36	3.16 ± 0.21	5.47 ± 0.28
LSD *p* = 0.05		1.05	0.93	0.83	1.02	0.59	0.79

A simple, statistically-valid approach to evaluating the impact of these barley *lpa* mutations on yield, and one that also reflects a good genetics design, is to conduct pair-wise “*T*-tests”, using the “LSD *p* = 0.05” value, to compare yields of sibling backcross lines that are either homozygous wild-type or homozygous mutant. Using this approach, if the difference in yield between wild-type and mutant sibling isolines is greater than the “LSD *p* = 0.05” value, one can conclude that the yield difference is statistically significant at the *p* = 0.05 level and is attributable to the allelic variant at the given *lpa* locus.

While variation due to isoline was of a lesser magnitude as compared with the main effects of Year and Location, it still was highly statistically significant. It is first important to note that no statistically-significant differences in yield were observed between the six wild-type isolines obtained after backcrossing and the original cultivar Harrington included as a control (Table 3 and Table 4). Yet differences are clear between the six wild-type isolines and Harrington on the one hand, and the cultivars Baronesse and Conrad on the other. This supports the experimental model that any observed differences in yield between a given wild-type and sibling *lpa* line are most likely attributable to the *lpa* allelic difference and not due to background genomic effects [5].

Of importance to this study are the two interactions of Year × Line and Location × Line. The Year × Line interaction was not statistically significant, indicating that isoline performances ranked similarly in each year. In contrast, the statistically significant Location × Line interaction indicates that isoline performance was differentially impacted by environment. This can be readily observed in the yield data when viewed over both years (Table 3 and Table 4): while the ranking of lines by yield might not have greatly differed between years, the magnitude of these differences in line yield varied. As a result, the large Year main effect adds power to the evaluation of the magnitude of differences in line yields between locations. 

Inspection of the yield data meaned over locations within each year indicates that one line, *Hvlpa*1-1, displayed grain yield equal to or greater than that of its sibling wild-type isoline in both years (Table 3 and Table 4). *Hvlpa*1-1 yielded as much or more than its sibling wild-type line in seven out of ten location/years, only yielding less in statistically-significant terms in one location/year (Rupert in 2010). In fact the results obtained for the Rupert 2010 location/year are anomalous. Despite being a presumed “non-stressful” location, all *lpa* isolines except for *lpa*4-1 yielded less than their wild-type siblings in that location/year, including the *lpa*1-1 isoline*.* We have no explanation for this anomalous result other than that it might be due to an unknown or unrecognized stress (or stresses) impacting the *lpa* lines more than the wild-type lines, or that it might indicate an example of unpredictability for *lpa* line yield, itself potentially important.

Despite this caveat, *Hvlpa*1-1 was similarly productive, as compared with its sibling wild-type isoline, in nearly all locations/years, including both stressful (Soda Springs in both years and Ashton in 2010) and non-stressful (Aberdeen, Idaho Falls, Rupert/2011) environments. A second mutation, *Hvlpa*4-1, yielded within 4% of its sibling wild-type isoline across all locations and years. Further, when yield comparisons are made between *lpa* and sibling wild-type lines when grown in the three least stressful, most productive environments (Aberdeen, Idaho Falls and Rupert), for three of the six *lpa* lines (*Hvlpa*1-1, *Hvlpa*3-1 and *Hvlpa*4-1), yields were consistently within 5% of the given sibling wild-type line.

Where the impact of the *lpa* trait on grain yield was most pronounced was in the most stressful, least productive environments, which in this experiment was Soda Springs, a “Dry-Land” production site where no irrigation was provided. Statistically-significant impacts on yield were observed for eight of the ten possible isoline/year pair-wise comparisons between mutant and wild-type at Soda Springs. The exceptions were *Hvlpa*1-1 in both years, and for *Hvlpa*3-1 in 2010 and *Hvlpa*4-1 in 2011.

In broad terms there does appear to be a correlation between the magnitude of grain phytic acid reduction conditioned by a given mutation and impact on yield. The consistently poorest performer was *lpa*-M955, which as a homozygote conditions a reduction in grain phytic acid that is >80%, and results in a ~20% reduction in grain yield, as compared with its sibling wild-type line. In the most stressful, poorest yield-potential location, Soda Springs, homozygosity for *lpa*-M955 results in a 30% to 50% reduction in yield. However, the correlation between the extent of grain phytic acid reduction conditioned by a mutant and its yield breaks down when one considers mutations, which condition more moderate reductions in grain phytic acid. The three best performers, *Hvlpa*1-1, *Hvlpa*3-1 and *Hvlpa*4-1 condition reductions in grain phytic acid ranging from ~40% to 60% (depending on environment), as compared with sibling wild-type lines, whereas the two mutations with intermediate performance, *Hvlpa*2-1 and *Hvlpa*-M640, condition reductions in the range of ~30%. Also, the best performer, *Hvlpa*1-1, is known to impact grain phytic acid accumulation in a tissue-specific manner, perturbing accumulation only in the endosperm. Therefore while the magnitude of grain phytic acid reduction conditioned by a mutation may be an important determinant of yield impact, gene function and tissue-specificity also play an important role.

## 3. Discussion

A previous study evaluated field performance of less advanced versions of four of these pairs of barley *lpa* near-isogenic lines, and was conducted at four sites in Idaho (USA) that include three used in the present study [5]. *Hvlpa*1-1, *Hvlpa*2-1 or *Hvlpa*3-1, conditioning seed phytic acid reductions of 30% to 60%, had yields similar to their wild-type sibling lines when grown in two relatively non-stressful production environments that utilized irrigation. *Hv-*M955, with greater than 90% reduction in seed phytic acid, yielded 13% less than its wild-type isoline in these environments. In the two non-irrigated, more stressful production environments included in the initial study, only *Hvlpa*1-1 displayed yields statistically similar to wild-type, but its yield was nevertheless 11% less than that recorded for its wild-type sibling. Furthermore, *Hvlpa*2-1 and *Hvlpa*3-1 each had 25% reduction in seed yield and *Hv-*M955 had nearly 35% reduction in seed yield. Thus in this initial study three out of four barley *lpa* isolines performed well in non-stressful, “high-yield potential” environments, but yield in more stressful environments was clearly reduced, and a trend for a yield reduction was observed even for the best performing *Hvlpa* line. This initial study was conducted over two years at two sites but only in one year at two additional sites.

The good field performance of *Hvlpa*1-1 lines observed in initial studies led to the breeding and registration of the first *lpa* cultivar of any crop species [6]. *Hvlpa*1-1 probably encodes a putative member of the sulfate transporter gene family [7]. Perturbation of this gene in barley also results in a second desirable crop seed phenotype, reduced seed total P [8]. Therefore this gene might prove to be an excellent target for engineering the ideal seed P phenotype, low-phytic acid and low total P, in other crops species.

In the present study performance comparisons were made using more advanced versions of the initial four sets of near-isogenic lines, obtained following one additional backcross, and an additional two sets of near-isogenic lines developed using *Hvlpa-*M640 and *Hvlpa*4-1. Furthermore, all six sets of isolines were evaluated across two years in five Idaho (USA) locations that greatly differ in crop yield potential. Confirming the results of the initial study [5], *Hvlpa*1-1 performed well in all locations, and had no detectable negative impact on yield.

A second mutation, *Hvlpa*4-1, resulted in yield losses of only 3.5%. Furthermore, when comparing yields in three relatively non-stressful production environments, at least three *lpa* mutations (*Hvlpa*1-1, *Hvlpa*3-1, and *Hvlpa*4-1) typically had yields similar to or within 5% of the wild-type sibling isoline. Thus while some *lpa* mutations do greatly or consistently impact field performance, a substantial fraction of barley *lpa* mutations appear to have little or no effect on yield. Therefore in the case of barley, *lpa* mutations can be readily identified that when simply incorporated into a cultivar result in adequately performing lines, at least in many production environments, even with no additional breeding for performance within the *lpa* line. Nevertheless, *lpa* line yield in more stressful environments remains problematic, as does consistency of performance for any given line in any environment. Therefore additional breeding via recurrent selection for field performance would still be needed to produce lines that are consistently high-yielding across diverse production environments (see below).

*Hvlpa*1-1 differs from the other three genotypes evaluated in the initial study [5] in that its recessive alleles result in a localized, tissue-specific block in phytic acid accumulation. Here we report that *Hvlpa*M640 is phenotypically similar to barley *lpa*1-1 in that it also appears to be endosperm-specific. However, the *Hvlpa*-M640 isoline did not perform as consistently well as did the *Hvlpa*1-1 isoline. This may indicate that it is not solely the endosperm-specificity of *Hvlpa*1-1 that results in its consistent performance, but rather that there are gene/function-specific differences impacting yield. Nevertheless, in contrast to the results of the present study yield trials indicate that a low-phytate barley cultivar produced using *Hv-*M640 has yields comparable with conventional cultivars in a wide variety of production environments [9]. Thus conventional genetics and breeding approaches can achieve at least one of the advantages provided by genetic engineering; genotypes in which blocks in the phytic acid pathways are restricted to specific tissues of the seed, such as the embryo or aleurone layer, thus minimizing the negative impact on whole plant performance and yield.

The only statistically valid experimental approach to attributing a quantitative difference in field performance, or in any trait, to a specific allelic variant of an *lpa* gene, is via comparisons using wild-type and mutant sibling near-isogenic lines. This is true regardless of the method used in generating the novel allelic variants that confer the *lpa* trait; whether the *lpa* trait is conferred by recessive alleles obtained following chemical or radiation mutagenesis, or obtained using any method that involves transformation or “genome modification”, methods that require tissue culture, regeneration or the use of selectable markers such as those conferring herbicide tolerance. A good example of this is “genome modification” [4], a powerful and highly valuable method that targets specific endogenous gene sequences and thus in theory avoids non-linked genomic change such as that resulting from chemical mutagenesis, or from non-homologous insertion/mutation resulting from earlier genetic modification methods. Genome modification has been used to target one of the maize (*Zea mays* L.) genome’s *IPK1* genes, which encodes an inositol-1,3,4,5,6-pentakisphosphate 2-kinase [4]. However, since tissue culture and regeneration can greatly impact genome expression and epigenetics in offspring [10], as can expression of reporter genes that confer herbicide resistance [11], assessing field performance using *lpa* progeny obtained via this method still first requires sufficient backcrossing, certainly a minimum of four to six backcrosses to selected recurrent parents, followed by isolation and evaluation of near-isogenic lines.

Even the use of near-isogenic lines has a potentially serious limitation. If there was no selection for performance during or after the incorporation of an allele from a divergent donor line into an elite line or cultivar, the resulting *lpa* or wild-type sibling lines might differ in yield due to inheritance of undesirable allelic variants at loci not subject to selection, or via “linkage-drag” of undesirable loci linked to the *lpa* locus in the donor parent [12]. This is particularly important because elite field performance reflects inheritance of favorable alleles, both genetic and epigenetic, at multiple loci throughout the genome. Thus simply incorporating an *lpa* allele via backcrossing, regardless of its source or method of induction, without some form of accompanying selection for performance, might result in lines with reduced performance that is not linked to the *lpa* trait or the *lpa* allele. Clearly, recurrent selection for field performance, within an *lpa* genotype over several generations, is required to obtain a line with favorable alleles genome-wide, both genetic and epigenetic, that retains the desired *lpa* phenotype and that also display elite performance. Despite this caveat, the use of near-isogenic lines, even without additional breeding, remains the most statistically appropriate approach to attributing a difference in yield or field performance to inheritance of an allelic difference at an *lpa* locus.

Few studies of field performance of *lpa* types have in fact made comparisons between truly near-isogenic sibling lines that, that to the best approximation available with current science, essentially differ only in the allelic variants of a single *lpa* locus. The first study reporting evaluation of the impact on crop performance or yield of an *lpa* type was Ertl *et al.* [12]. In this study, the maize *lpa*1-1 allele (*Zmlpa*1-1), originally isolated in a “Flint corn-like” genetic background referred to as “Early ACR” [13], was backrossed to the BC_3_ (four crosses to the recurrent parent inbred line), into a sufficient number of diverse elite maize “Dent corn” inbred lines to produce a total of 14 pairs of near-isognenic hybrids, each pair consisting of a wild-type and *Zmlpa*1-1 version. The performance evaluation of these pairs of maize hybrids consisted of studies conducted in one year, at three locations with two replications, in central Iowa, USA. In six of the 14 near-isogenic pairs no statistically-significant effect of the *Zmlpa*1-1 allele on yield was observed. In eight of the 14 near-isogenic pairs a yield loss due to the *Zmlpa*1-1 was observed, and overall the yield loss was 6%, as compared with wild-type. Thus this first report of performance evaluation of low-phytate versions of a crop reported results similar to those reported here. However, while ground-breaking, this first study had inherent limitations. The production environments and practices were similar with no clearly stressful or low-productivity environments, and replication was minimal. More importantly the Flint and Dent corn genetic backgrounds used greatly differ, and only four crosses to the recurrent parent (the Dent corn inbred lines) were completed. Therefore the resulting pairs of lines can only be considered “early generation” near-isogenic lines, with residual genomic contribution of the very divergent donor parent of approximately 6.25%. In the present study the *lpa* mutations were isolated in the same genetic background that they were subsequently backcrossed to, cv. Harrington. Thus in this case even “early-generation” backcross lines, such as the *Hvlpa*4-1 and *Hvlpa-*M640 lines, are more genetically similar to their wild-type sibling lines than those evaluated in the Ertl *et al.* study [12].

A third series of studies that evaluated field performance of *lpa* types using near-isogenic lines addressed issues of germination and field emergence in *lpa* soybean [*Glycine max* (L.) Merr.] types [14,15,16]. Germination and emergence is reduced in many soybean *lpa* genotypes, and the effect is highly pronounced when seed is produced in tropical *versus* temperate environments, a phenomenon referred to as the “seed source effect” [14]. When low-phytate soybean lines were produced via incorporation of recessive alleles of the soybean MIPS-encoding gene (the LR33 mutations) [17], or the *pha1* and *pha2* alleles first isolated as the M153 mutant [18], and seed was produced in a tropical environment, subsequent field emergence was greatly reduced when compared to that of seeds of the same genotype when tested seed was produced in a more moderate season in Puerto Rico, or in a temperate region [14,16]. While the extreme effects on germination and emergence that maturation in a tropical environment produces has not been overcome via subsequent breeding, evaluation of BC_4_-derived near-isogenic lines indicated that selection could identify lines with improved performance [16].

The studies with soybean and barley *lpa* genotypes illustrate the potential of classical genetics and breeding approaches for overcoming agronomic problems associated with the *lpa* trait. Selection within lines segregating for a given *lpa* allele can identify lines with enhanced agronomic performance [15]. Identification of genes that, if perturbed, have little impact on agronomic performance, such as barley *lpa*1 and M640 [6,9], or soybean *lpa*-ZC-2 [19], can lead to low-phytate cultivars with superior agronomic performance.

## 4. Experimental Section

### 4.1. Isoline Pair Development and Cultivar Controls

Each of six *lpa* mutations were crossed and subsequently backcrossed to varying degrees to the cultivar Harrington in which they were originally isolated. BC_x_F_2_ progenies were obtained following self-pollination of a given BC_x_F_1_, and sibling homozygous wild-type and homozygous mutant plants were identified. These were then used for increases to provide seed for the present studies. Seed used in the 2010 yield trial were: BC_5_F_4_ (*Hvlpa*1-1 and its wild-type sibling line); BC_4_F_4_ (*Hvlpa*2-1, *Hvlpa*3-1, and *Hvlpa*-M955 and their wild-type sibling lines); F_4_ (*Hvlpa*4-1 and *Hvlpa*-M640). Seed used in the 2011 yield trial were advanced further generations as follows: BC_5_F_5_ (*Hvlpa*1-1 and its wild-type sibling line); BC4F5 (Hvlpa2-1, Hvlpa3-1, and Hvlpa-M955 and their wild-type sibling lines); BC1F3 (Hvlpa4-1 and *Hvlpa*-M640). Since each mutation was first isolated in the genetic background represented by the cultivar Harrington, then crossed or backcrossed to the same cultivar, following which sibling plants either homozygous wild-type or homozygous mutant were increased, each pair of sibling lines can be considered near-isogenic. For confirming the *lpa* genotype and phenotype of a given plant or line a microtitre plate assay for seed inorganic P was used (Supplemental Figure 1): homozygous wild-type lines typically have relatively low levels of inorganic P (˂0.5 mg·g^−1^), whereas homozygous *lpa* lines typically have greatly elevated seed inorganic P (>1.0 mg·g^−1^ in most cases). This “high seed inorganic P” phenotype provides a simple assay visual inspection of which is sufficient to genotype lines and test for homogeneity (Supplemental Figure 1).

In addition to the six pairs of near-isogenic lines, three barley cultivars were included in the field trials as controls. These were: (1) Harrington, a “2-rowed malting barley” that represents the genetic background in which these mutations were isolated; (2) Baronesse, a “two-rowed feed barley” that is observed to be one of the highest yielding barleys when grown in the Pacific Northwest region; and (3) Conrad, a “2-rowed malting barley” that has displayed higher yields than Harrington when produced in the Pacific Northwest region.

### 4.2. Test Sites

The six pairs of isolines, and three cultivar controls (see below) were grown in field trials in two consecutive years (2010 and 2011) at five locations in Idaho that represent diverse environments (soil types, elevation, growing season, temperature and water availability) and diverse production practices (irrigation *versus* non-irritation, planting density). These location differences result in clearly diverse yield capacities. These test sites were: (1) Aberdeen Research and Extension Center, Coordinates 42°57'47.23'' N, 112°49'9.31'' W, elevation 1341 M, soil type Declo fine sandy loam with sprinkler irrigation; (2) Ashton, Coordinates 44°04'53.96'' N, 111°19'8.16'' W, elevation 1603 M, soil type #92 rin silt loam, sprinkler irrigation; (3) Idaho Falls, Coordinates 43°28'33.15'' N, 112°07'14.93'' W, elevation 1430 M, soil type #22 Pancheri silt loam, sprinkler irrigated; (4) Rupert, Coordinates 42°43'14.95'' N, 113°30'36.13'' W, elevation 1297 M, soil type #36 Sluka silt loam, sprinkler irrigation; and (5) Soda Springs, Coordinates 42°42'13.10'' N, 111°36'30.80'' W, elevation 1826 M, non-irrigated “Dry-land”.

Prevailing agronomic practices were used at all sites. Seed for planting was not pre-treated with fungicide. Planting and harvest dates varied between locations reflecting prevailing local conditions in each year. Planting density was 90 kilos of seed·ha^−1^ at irrigated sites, and 68 kilos of seed·ha^−1^ at non-irrigated sites. Row spacing was 18 cm for all sites. Harvested plots at all locations except for Aberdeen were 1.5 M by 3.0 M. At Aberdeen plots were 1.5 M by 2.8 M. All entries were replicated 4 times at each location in a randomized complete block (RCB) design. Nitrogen fertilizer in irrigated locations was managed according to the following methodology: yield goals were set for each class at each location using historical yield data. These yield goals were used to calculate optimal fertility amounts according to the following methods: nitrogen needed to produce 1.7 times the yield goal. Details of fertilizer application and meteorological data (temperature and rainfall) are available upon request.

### 4.3. Data Collection

Agronomic data collected includes grain yield (Mg·ha^−1^), test weight (g 30 L^−1^), plant height, “heading date” as the date when 50% of heads are fully emerged, and lodging as a visually determined percentage of each plot that is not standing straight at harvest. Additional agronomic data not reported here are available upon request.

Seed P fractions were assayed as follows. Samples of mature seeds were ground with a coffee grinder. Total P was determined following wet-ashing of aliquots of flour (150 mg) and colorimetric assay of digest P [20]. For determination of seed phytic acid P and inorganic P, aliquots of flour (50 mg) were extracted in 1.0 mL of extraction media (0.2 M HCL:10% Na_2_SO_4_) in 1.5 mL Eppendorf tubes overnight at 4 °C with shaking. Extracts were centrifuged (4500 RCF, 20 min). Supernatant phytic acid P was determined using a modification of the method as described [21] and supernatant inorganic P was determined colorimetrically [20].

### 4.4. Statistical Analyses

Statistical analyses were conducted using the General Linear Models procedures developed by the SAS Institute (Raleigh, NC, USA).

## 5. Conclusions

The results presented here demonstrate that in the case of barley, *lpa* mutations can readily be identified that even with no additional breeding have little or no effect on yield. This conclusion is supported by a very stringent test; a multi-year, multi-location yield trial that compared yields between six sets of sibling near-isogenic lines. This represents the most thorough field trial of *lpa* crop variants published to date. Thus the perception that the *lpa* trait is obligately correlated with significantly reduced yield should be moderated; many *lpa* mutations do greatly impact plant and seed performance and thus have a significant impact on yield, but some have little or no effect on yield and can be used to breed crops with substantially enhanced nutritional quality. However it is also possible that the relative lack of impact on yield observed here simply reflects a difference between barley and other cereal and legume crop species: that the relatively small impact on yield of barley *lpa* mutations is somehow a unique characteristic of barley. 

## Supplementary Files

Supplementary File 1
