# Supplementary material for: A Substantial Fraction of Barley (Hordeum vulgare L.) Low Phytic Acid Mutations Have Little or No Effect on Yield across Diverse Production Environments
Source: Plants (Basel). 2015 Apr 29;4(2):225–39. doi: 10.3390/plants4020225 (PMC4844328; doi:10.3390/plants4020225)
Supplement: Supplementary File 1 [file plants-04-00225-s001.pdf]

Supplementary Information

Supplementary Methods: Table S1

Seed of the "two-rowed" malting barley *cv.* Harrington [1] were treated with sodium azide using a modification of the method as described [2], and advanced to the M_2 generation. M_3 seed sampled from a total of 2601 M_2s were screened with the single-seed "high inorganic P" (HIP) test as described [3]. Wild-type barley seeds typically contain ≤0.7 mg inorganic P g^{-1}. Seed testing for >1.0 mg inorganic P g^{-1} were deemed "HIP". Direct-descendant lines (M_6 to M_8) that appeared homozygous for selected putative mutants were planted in a summer nursery in 2004 at the University of Idaho's Aberdeen Research and Extension Center. Non-mutagenized cv. Harrington was included as a control. Seeds were harvested from individual plants and stored at 6 ℃ and 25% relative humidity. Subsequent analyses revealed that of the 23 putative mutants, one was a false positive and two (MAz104 and MAz237), while apparently heritable, had not yet been obtained as homozygotes. The remaining 20 were the subject of further analyses.

Two plants (replicates) representing each line were selected for analysis, and each replicate was analyzed in duplicate. Seed P fractions were determined in whole-seed samples, and following a simple dissection [4], in "germ-half-seeds" and "endosperm-half-seeds". For whole-seed analyses, samples of 50 to 100 mature seeds were dried for 48 h at 60 ℃ and weighed. For half-seed analyses, samples of 20 seeds were cut in half by hand at the mid-way point between base and apex, the basal half containing the germ, and the apical half largely consisting of endosperm and aleurone. Whole-seed and half-seed samples were milled to pass through a 20-mesh (~0.853 mm) screen, and stored in a desiccator until analysis. Seed total P was determined following wet-ashing of aliquots of tissue (typically 150 mg) and colorimetric assay of digest P [3]. The ferric-precipitation method was used to determine total, acid-soluble inositol phosphates, referred to here as phytic acid P [5]. HPLC analyses indicated that in most genotypes >90% of total seed inositol phosphate consists of phytic acid (inositol hexakisphosphate), with the remainder consisting of less highly phosphorylated inositol phosphates such as inositol tetrakisphosphate or pentakisphosphate (data not shown). Thus use of the term "phytic acid P" for total inositol Ins phosphate is accurate in most cases. Aliquots of tissue (typically 0.5 to 1.0 gm) were extracted in 0.4 M HCl:0.7 M Na_2SO_4. Phytic acid P was obtained as a ferric precipitate, wet-ashed and assayed for P as in the total P analysis. Phytic acid P is expressed in terms of its P (atomic weight 31) content to facilitate comparisons between seed P fractions. Seed inorganic P was determined colorimetrically following extraction of tissue samples (typically 0.5 g in wild-type seeds and 0.15 g in mutant seeds) in 12.5% (*w/v*) TCA:25 mM $MgCl_2$. Statistical analyses were conducted using Statistical Analysis Systems (SAS) Software 9.2, SAS Institute Inc., Cary, NC, USA.

Table S1. Seed dry weight and seed phosphorus (P) fractions in 21 barley lines consisting of the wild-type control (*cv*. Harrington) and twenty mutants that display the "high inorganic P" seed phenotype. Seed was obtained from the 2004 Aberdeen, Idaho nursery. Please see Supplementary Methods for details of whole-grain and "half-seed" analysis and other methods used.

Cultivar or Mutant Line	Seed Dry Weight	Total P				Phytic Acid P				PAP/ Tot P	Inorganic P	(PAP + Inorg. P)/ Total P
		Whole Grain	Half-Seed Analysis			Whole Grain	Half-Seed Analysis					
			Endo-Sperm	Germ	Endo/ Germ		Endo-Sperm	Germ	Endo/ Germ			
	mg seed^{-1}	mg g^{-1}	mg g^{-1}	mg g^{-1}		mg g^{-1}	mg g^{-1}	mg g^{-1}			mg g^{-1}	
Wild-Type	52.9	4.54	4.04	4.50	0.90	2.75	2.73	3.07	0.89	0.60	0.61	0.74
**Chromosome 2H Linkage Group**												
*Hvlpa*1-1 (M422)	43.9	3.70	3.08	4.38	0.70	1.26	0.54	1.94	0.28	0.34	1.54	0.76
M281	41.6	3.77	2.67	3.88	0.69	1.30	0.38	1.65	0.23	0.35	1.69	0.79
M882	47.4	3.84	2.81	4.14	0.68	1.30	0.51	1.83	0.28	0.34	1.54	0.74
M1154	45.7	3.70	2.66	4.24	0.63	1.31	0.50	1.56	0.33	0.36	1.43	0.74
M1580	43.8	3.97	3.05	4.94	0.62	1.46	0.53	2.00	0.26	0.37	1.54	0.76
M1673	43.9	3.72	2.87	4.38	0.66	1.53	0.62	2.16	0.29	0.41	1.37	0.78
M2002	45.8	4.02	2.59	4.37	0.59	1.43	0.48	2.18	0.22	0.36	1.40	0.70
M2172	42.6	3.68	2.54	4.20	0.60	1.37	0.60	1.98	0.30	0.37	1.30	0.73
**Chromosome 7H Linkage Group**												
Hvlpa 2-1 (M1070)	46.5	4.85	4.38	4.69	0.93	1.03	0.89	0.98	0.91	0.21	2.75	0.78
M640	38.9	4.19	3.13	4.98	0.63	1.49	0.65	2.27	0.28	0.36	1.70	0.76
**Chromosome 1H Linkage Group**												
Hvlpa 3-1 (M635)	43.5	4.80	4.14	4.64	0.89	1.03	0.87	1.09	0.81	0.21	2.67	0.77
M955	39.4	5.00	4.62	5.17	0.89	0.24	0.16	0.22	0.70	0.05	3.46	0.74

Table S1. *Cont.*

Cultivar or Mutant Line	Seed Dry Weight	Total P				Phytic Acid P				PAP/ Tot P	Inorganic P	(PAP + Inorg. P)/ Total P
		Whole Grain	Half-Seed Analysis			Whole Grain	Half-Seed Analysis					
			Endo-Sperm	Germ	Endo/ Germ		Endo-Sperm	Germ	Endo/ Germ			
	mg seed^{-1}	mg g^{-1}	mg g^{-1}	mg g^{-1}		mg g^{-1}	mg g^{-1}	mg g^{-1}			mg g^{-1}	
**Chromosome 4H Linkage Group**												
*Hvlpa*4-1 (M593)	46.9	4.57	3.80	4.26	0.89	1.28	0.94	1.29	0.73	0.28	2.18	0.76
M678	29.2	5.22	4.78	5.32	0.90	0.18	0.13	0.18	0.75	0.04	3.76	0.76
M889	52.2	3.84	3.53	3.63	0.98	1.70	1.35	1.59	0.84	0.44	1.00	0.70
M1572	51.4	4.75	4.54	5.19	0.88	2.14	1.54	1.78	0.87	0.45	1.90	0.85
M1954	46.8	4.73	4.08	4.32	0.94	2.40	2.28	2.19	1.04	0.51	1.29	0.78
**Others Mutants of presently unknown inheritance and map position.**												
M499	44.4	4.77	4.05	4.54	0.89	2.40	2.33	2.34	1.00	0.51	1.08	0.73
M2080	38.8	4.70	4.43	4.41	1.00	1.93	1.91	1.66	1.15	0.41	1.71	0.77
MAZ423	49.2	5.61	4.94	5.50	0.90	3.17	2.99	3.14	0.95	0.56	1.12	0.76
LSD 0.05	7.3	0.47	0.50	0.59	0.07	0.40	0.21	0.24	0.09	0.09	0.38	NA
F Value	4.55 ***	13.2 ***	22.3 ***	5.63 ***	39.3 ***	28.3 ***	142 ***	80.6 ***	104 ***	21.3 ***	38.6 ***	0.80 ^{NS}

Supplementary Methods: Tables S2 and S3

The barley cultivars "Morex" [6] and "Steptoe" [7] are widely used in barely mapping and genomics research and represent distinct germplasm pools as compared with *cv.* Harrington [8]. Therefore for chromosomal mapping, F_2 populations were derived from crosses between each Harrington-derived seed P mutant and both Morex and Steptoe. F_2's were grown at the Aberdeen Research and Extension Center in 2002, and in subsequent greenhouse nurseries, to provide populations ranging in number from approximately100 to 200 recombinants. F_3 progeny seed for each F_2 plant were harvested and stored until analysis.

Segregation analysis for each seed P mutation was conducted by testing individual F_3 seed for the "high inorganic P" typical of each mutation. As homozygotes these mutations condition increases in seed inorganic P as compared with a heterozygote or homozygous wild-type seed that are typically large enough to permit clear-cut scoring on an individual kernel basis [9,10]. Typically a minimum of 30 individual kernels from each F_3 progeny were tested. If all or nearly all (>27) F_3 seed displayed the seed inorganic P phenotype typical of the parental mutant, the F_2 parent plant was scored as homozygous mutant. If all F_3 seed were scored as wild-type, then the F_2 parent plant was scored as homozygous wild-type. If segregation for the HIP phenotype was observed in the F_3 (three or more kernels per 30 F_3 kernels scored as mutant/high inorganic P, with the remainder intermediate or wild-type/low inorganic P), then the parent F_2s were scored as heterozygotes. If the result of the initial round of seed testing was inconclusive, additional F_3 seed testing was conducted. Data were collected using either images of colorimetric reactions or digital readings from Synergy™ 2 Multi-Detection Microplate Reader (BioTEK, Winooski, VT, USA). For mapping purposes, $F_{2\text{–}3}$ families homozygous for either mutant or wild-type alleles from each segregating population were used to simplify the mapping score. Leaf samples were collected, immediately frozen in liquid N_2 and stored at −80 ℃.

PCR-based markers linked to each of the three previously-known *Hvlpa* loci and the barley genome's single gene encoding *myo*-Inositol-3-P_1 synthase (MIPS) [11] were selected based on the previously published data [9–11]. For the *Hvlpa*1 locus on barley chromosome 2H, aMSU21 was identified as a closely linked STS-PCR marker [9,12]. New primer pairs for the aMSU21 locus were designed; aMSU21 Forward (5'-tggtctttcatgtacctacc-3') and aMSU21 Reverse (5'-tgtgtcatcaagcacaacca-3'). The newly designed primer pairs amplify a single strong 435 bp PCR product from Steptoe and Morex that is slightly shorter than the original 449 bp product, and about 200 bp bigger PCR products from Harrington and its derived mutations. The original primer pairs of aMSU21 detected two major fragments from each cultivar [12] while our modified primer pairs detected one major fragment from each genotype (data not shown). Thus the modified marker simplified the genotype scoring. The barley MIPS locus on chromosome 4H was first mapped close to the BCD453 marker [11]. The EBmac701 SSR marker was identified as flanking BCD453B (Barley Consensus Map 2005, GrainGenes, http://wheat.pw.usda.gov/cgi-bin/graingenes/report.cgi?class=mapdata&name=Barley,+Consensus+ 2005,+SNP) [11]. The EBmac701 primer pairs listed in GrainGenes (Forward, 5'-atgatgagaactcttcaccc-3' and Reverse, 5'-tggcactaaagcaaaagac-3') amplified a single strong PCR product. The polymorphic patterns were clearly scored as a ~150 bp product in Harrington and slightly smaller, but clearly scorable fragments in Steptoe and Morex (data not shown).

For the *Hvlpa*2 locus on barley chromosome 7H, the previously described flanking RFLP markers were MWG2301 and ABC310b [9]. Bmag0120 was chosen based on its close location (Barley shedar2 map, Grain Genes) to ABC310b that was loosely linked to *Hvlpa*2-1 [9]. The Bmag120 primers listed in GrainGenes (Forward, 5'-atttcatcccaaaggagac-3' and Reverse, 5'-gtcacatagacagttgtcttcc-3') amplified a ~250 bp single product in Harrington and a slightly smaller product in Steptoe. For the *Hvlpa*3 locus on barley chromosome 1H, the RFLP probes cWMG706 and ABG702B were shown to flank the locus and the closely-linked LP75 sequence-specific ISSR primer was developed [10]. Since LP75 requires ClaI digestion of PCR products to detect polymorphism, the Bmag382 SSR marker, closely linked to cWMG706 (Igri × Franka map, GraineGenes) was chosen for this experiment. The Bmag382 primer pairs listed in GrainGenes (Forward, 5'-tgaaacccatagagagtgaga-3' and Reverse, 5'-tcaaaagtttcgttccaaata-3') amplified a strong single PCR product in all barley cultivars in this experiment. The PCR product from Harrington is slightly above the 100 bp size marker distinguishing the slightly smaller fragments from Steptoe and Morex (data not shown).

Leaf samples collected from $F_{2\text{–}3}$ plants were lyophilized prior to DNA extraction with cetyl trimethyl ammonium bromide (CTAB). Pulverized leaf tissue in a 96-well format was extracted with the protocol utilized by [13]. The only modification to the procedure was the use of chloroform without isoamyl alcohol.

PCR reactions were set up in a 96-well format. Each 25 μL reaction contained 50 ng of template DNA, 2 μL of each primer (10 μM), 2.5 μL of 10× buffer containing 1.1 mM Mg, 1 μL of dNTPs with 2.5 mM concentration for each nucleotide, and 1 unit of Taq polymerase (RedTaq, Sigma). The PCR program was: 94 °C for 3 min followed by 39 cycles of 94 °C for 30 s, 50 °C for 30 s, and 72 °C for 1 min, followed by a 4 °C hold. PCR reaction products were analyzed on 3% Agarose SFR high resolution gels (Amresco, Solon, OH, USA) stained with ethidium bromide. A 100 bp DNA ladder (Bio-Rad, Richmond, CA, USA) was used as a size marker in the same gel.

The PCR fragments were scored for each individual F_2 plant from segregating populations and compared to the genotype patterns of both parents. Mutant or wild-type scores were recorded for all the plants used. The genotype scores were then compared to the seed inorganic P phenotype as determined in F_3 seeds derived from F_2 plants described above.

Table S2. Segregation ratios observed in barley *low phytic acid* (*lpa*) F_2 mapping populations. Please see Supplementary Methods for details of analyses.

Parental Cross	F_2 Progeny Genotypes [a]			Chi-Square (1:2:1) [b]	Chi-Square (3:1) [c]
	Lpa/Lpa	***Lpa/lpa***	***lpa/lpa***		
M 281 × Steptoe	17	36	19	0.2	0.1
M 499 × Steptoe	16	22	4	6.9 *	5.3 *
M 593 × Steptoe	22	42	23	0.3	0.2
M 640 × Steptoe	22	44	16	1.3	1.3
M 678 × Steptoe	22	31	19	1.7	0.1
M 882 × Steptoe	22	51	23	0.6	0.2
M 889 × Morex	45	33	5	42 *	15.9 *
M 1154 × Steptoe	24	49	20	0.6	0.7
M 1572 × Steptoe	24	42	19	0.8	0.6
M 1580 × Steptoe	23	34	28	3.9	2.8
M 1673 × Steptoe	26	42	19	2.7	1.8
M 1954 × Steptoe	23	35	10	5.0	3.9 *
M 2002 × Steptoe	12	45	10	8.0 *	3.7
M 2172 × Morex	28	40	8	10.8 *	7.8 *

* Significant deviation from expected genotype ratio ($\alpha = 0.05$); [a] *Lpa*/*Lpa*, *Lpa*/*lpa*, and *lpa*/*lpa* indicates homozygous wild-type, heterozygous and homozygous mutant, respectively; [b] Critical value for Chi-square distribution with tail area probability of 0.05 and $df = 2$ is 5.99; [c] Critical value for Chi-square distribution with tail area probability of 0.05 and $df = 1$ is 3.84.

Table S3. Linkage analyses of SSR markers to barley *low phytic acid* mutations. Please see Supplementary Methods for details of analysis.

Mutation	DNA Marker Linkage							
	aMSU21		EBmac701		Bmag0120		Bmag382	
	Rec. [a]/ Total	Genetic Distance (cM)	Rec./ Total	Genetic Distance (cM)	Rec./ Total	Genetic Distance (cM)	Rec./ Total	Genetic Distance (cM)
M 281	3/160	1.9						
*Hvlpa*1-1	8/142	5.6						
M 882	1/41	2.4						
M 1154	2/42	4.8						
M 1580	2/58	3.4						
M 1673	1/51	2.0						
M 2002	1/138	1.0						
M 2172	1/63	2.0						
M 640	28/51	54.9	26/51	51.0	8/51	15.7	27/51	52.9
M 1954			12/41	29.3				
M 593			18/57	31.6	33/57		41/57	
M 1572			12/50	22.0				
M 889	25/34	73.5	10/34	29.4			20/34	58.8
M 678	46/66	69.7	16/66	24.2	47/66	71.2	47/66	71.2
M 499	22/28	78.6	17/28	60.7	16/28	57.1	17/28	60.7

[a] Rec. = recombinants in the tested population. The raw scored data is presented here.

Supplementary Methods: Figure S1

Forty seed were randomly sampled from the seed bulk representing each isoline. Individual seed were crushed with a lab press, extracted overnight in 250 µL 0.4 M HCl, and 10 µL extract assayed for seed inorganic P using a microtitre plate assay as described [5]. The Colorimertric Standards No. 1 through 5 contain 0.0, 0.15, 0.46, 0.93, and 1.39 µg inorganic P per well. Wild-type barley seed typically contain ≤0.5 mg inorganic P g^{-1} dry weight, and single-seed assays usually result in tests visually similar to Standards 1 through 3. Homozygous *lpa* seed typically contain ≥1.0 mg inorganic P g^{-1}, and single-seed assays usually result in tests similar to Standards 4 and 5.

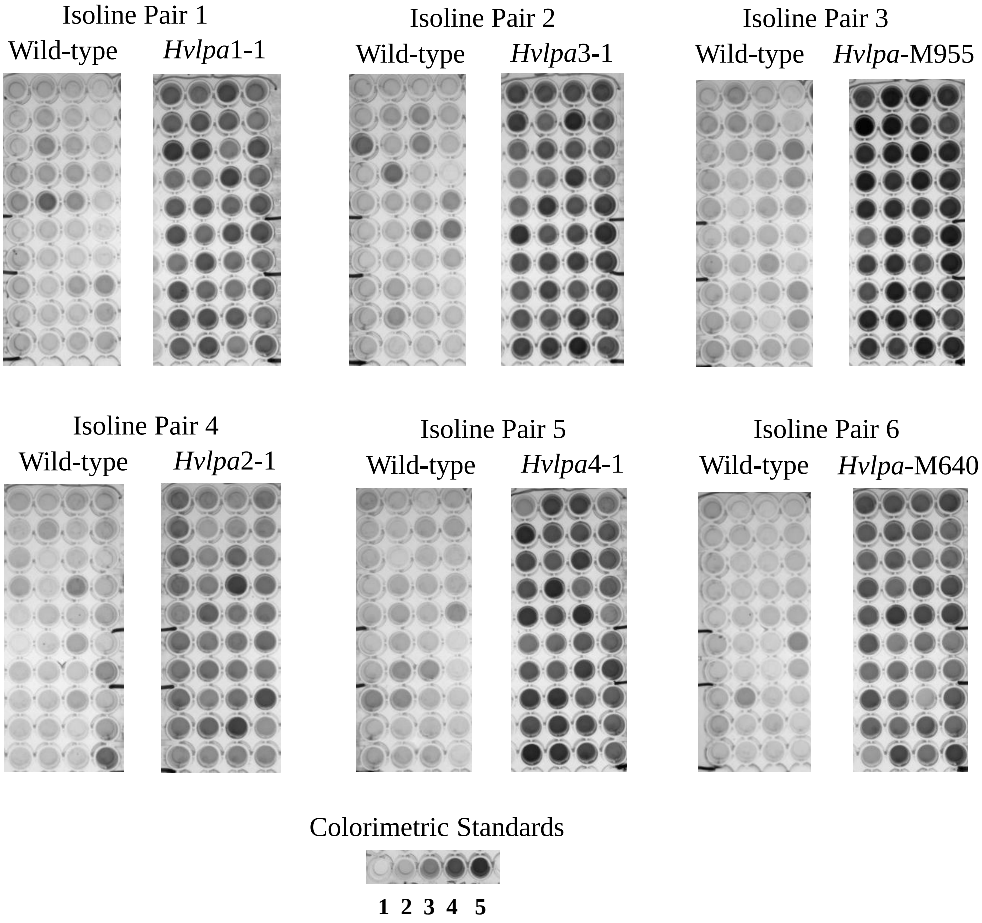

Figure S1. Tests for homozygosity and homogeneity in barley *low phytic acid* near-isogenic lines. Single-seed tests were conducted for inorganic P in seed produced by pairs of barley near-isogenic lines, each pair consisting of a sibling wild-type or homozygous mutant line. The Colorimertric Standards No. 1 through 5 contain 0.0, 0.15, 0.46, 0.93, and 1.39 µg inorganic P per well. Wild-type barley seed typically contain ≤0.5 mg inorganic P g^{-1} dry weight, and single-seed assays usually result in tests visually similar to Standards 1 through 3. Homozygous *lpa* seed typically contain ≥1.0 mg inorganic P g^{-1}, and single-seed assays usually result in tests similar to Standards 4 and 5.

Supplementary References

1. Harvey, B.L.; Rossnagel, B.G. Harrington barley. *Can. J. Plant Sci.* **1984**, *64*, 193–194.
2. Nilan, R.A.; Sideris, E.G.; Sander, C.; Konzak, C.F. Azide—A potent mutagen. *Mutat. Res.* **1973**, *17*, 142–144.
3. Dorsch, A.J.; Cook, A.; Young, A.K.; Anderson, N.J.; Bauman, T.A.; Volkmann, J.C.; Murthy, P.N.P.; Raboy, V. Seed phosphorus and inositol phosphate phenotype of barley low phytic acid genotypes. *Phytochemistry* **2003**, *62*, 691–706.
4. Raboy, V.; Cichy, K.; Peterson, K.; Reichman, S.; Sompong, U.; Srinives, P.; Saneoka, H. Barley (*Hordeum vulgare* L.) *low phytic acid* 1-1: An endosperm-specific determinant of seed total phosphorus. *J. Hered.* **2014**, *105*, 656–665.
5. Raboy, V.; Gerbasi, P.F.; Young, K.A.; Stoneberg, S.D.; Pickett, S.G.; Bauman, A.T.; Murthy, P.P.N.; Sheridan, W.F.; Ertl, D.S. Origin and seed phenotype of maize *low phytic acid* 1-1 and *low phytic acid* 2-1. *Plant Physiol.* **2000**, *124*, 355–368.
6. Rasmusson, D.C.; Wilcoxson, R.D. Registration of Morex barley. *Crop Sci.* **1979**, *19*, 293.
7. Muir, C.E.; Nilan, R.A. Registration of Steptoe barley. *Crop Sci.* **1973**, *13*, 770.
8. Matus, I.A.; Hayes, P.M. Genetic diversity in three groups of barley germplasm assessed by simple sequence repeats. *Genome* **2002**, *44*, 1095–1106.
9. Larson, S.R.; Young, K.E.; Cook, A.; Blake, T.K.; Raboy, V. Linkage mapping two mutations that reduce phytic acid content of barley grain. *Theor. Appl. Genet.* **1998**, *12*, 141–146.
10. Roslinsky, V.; Eckstein, P.E.; Raboy, V.; Rossnagel, B.G.; Scoles, G.J. Molecular marker development and linkage analysis of low phytic acid barley (*Hordeum valgare*) mutant lines. *Mol. Breed.* **2007**, *20*, 323–330.
11. Larson, S.R.; Raboy, V. Linkage mapping of maize and barley myo-inositol 1-phosphate synthase DNA sequences: Correspondence with a low phytic acid mutation. *Theor. Appl. Genet.* **1999**, *99*, 27–36.
12. Shin, J.S.; Chao, S.; Corpuz, L.; Blake, T.K. A partial map of the barley genome incorporating restriction fragment length polymorphism, polymerase chain reaction, isozyme, and morphological marker loci. *Genome* **1990**, *33*, 803–810.
13. Jackson, E.W.; Avant, J.B.; Overturf, K.E.; Bonman, J.M. A quantitative assay of *Puccinia coronata* f. sp. *avena* DNA in *Avena sativa*. *Plant Dis.* **2006**, *90*, 629–636.

© 2015 by the authors; licensee MDPI, Basel, Switzerland. This article is an open access article distributed under the terms and conditions of the Creative Commons Attribution license (http://creativecommons.org/licenses/by/4.0/).
